# Diminished verbal ability among children conceived through ART with exposure to high serum estradiol in utero

**DOI:** 10.1007/s10815-020-01835-1

**Published:** 2020-06-09

**Authors:** Cheng-Liang Zhou, Gu-Feng Xu, Qian Yang, Hui-Hui Wang, Meng-Xi Guo, Yi-Meng Xiong, Xiao-Yan Guo, Min Hou, Lu-Yang Jin, Jian-Zhong Sheng, Lin He, Li Jin, He-Feng Huang

**Affiliations:** 1 International Peace Maternity and Child Health Hospital, School of Medicine, Shanghai Jiao Tong University, Huashan Rd. 1961, Shanghai, 200030 China; 2 Shanghai Key Laboratory of Embryo Original Diseases, Shanghai, China; 3 Department of Reproductive Endocrinology, Women’s Hospital, School of Medicine, Zhejiang University, Hangzhou, China; 4 Key Laboratory of Reproductive Genetics, Ministry of Education, Zhejiang University, Hangzhou, China; 5 Department of Reproductive Endocrinology, Zhejiang Provincial People’s Hospital, Hangzhou, China; 6 Department of Pathology and Pathophysiology, School of Medicine, Zhejiang University, Hangzhou, China; 7 Bio-X Institutes, Shanghai Jiao Tong University, Shanghai, China

**Keywords:** Assisted reproductive technology, Estradiol, Intelligence quotient, Verbal ability

## Abstract

**Purpose:**

Higher serum estradiol levels occur in women undergoing assisted reproductive technology (ART) owing to ovarian stimulation. Here, we investigated the association between maternal serum estradiol levels and the intellectual development of offspring conceived with ART.

**Methods:**

A total of 204 singletons born after fresh embryo transfer were recruited for this cohort study. Among them, 102 children were born from mothers with high serum estradiol levels (> 12,000 pmol/L) on the day that human chorionic gonadotropin was administered. Another 102 children, matched by gestational age and age of the children, were recruited as controls from mothers with low serum estradiol (≤ 12,000 pmol/L). The Wechsler Preschool and Primary Scale of Intelligence was used to evaluate the intellectual development of the children.

**Results:**

Children from mothers with higher serum estradiol levels scored lower in the verbal intelligence quotient (IQ) tests and verbal comprehension than children whose mothers had lower estradiol levels. The main difference between the two groups was in verbal subtests including information, vocabulary, and sorting. Partial correlation analysis revealed that the logarithm of maternal serum estradiol level negatively correlated with verbal IQ, performance IQ, and full scale IQ.

**Conclusion:**

Our data demonstrate that a high maternal serum estradiol level may negatively associate the verbal ability of children conceived via ART.

## Introduction

Accumulating research suggests that exposure of a fetus to an abnormal environment in the uterus can cause chronic disease in later life [1–4], as has been observed in both human populations and animal models [4–9].

Assisted reproductive technology (ART) was pioneered in 1978 and is now widely used; however, its short- and long-term effects on offspring are not fully understood. Superovulation has been the conventional strategy for ART, but multi-follicle development also generates excessively high hormone levels in maternal serum [10]. Women who undergo fresh embryo transfer, in comparison with frozen embryo transfer and spontaneous conception, experience elevated estradiol levels throughout early pregnancy [10, 11]. Studies have shown that exposure of a fetus to high maternal serum estradiol is associated with low birth weight, dyslipidemia, and dysfunction of the thyroid and cardiovascular system in offspring [12–15]. Moreover, offspring of mothers who suffered from ovarian hyper-stimulation syndrome (OHSS) have reduced intelligence, which has been associated with supra-physiological levels of maternal serum estradiol [16].

Estradiol has a remarkable effect on neuroendocrine system development and associated behaviors. During prenatal life, fetal brain development encounters waves of steroid hormones arising from the mother, placenta, and the developing fetal gonads and adrenals, and any mistiming or inappropriate hormone levels may affect the fetus and result in developmental dysfunction [17]. However, the impact of high maternal serum estradiol on the intellectual development of offspring is not well understood. In this study, we explored the association between high maternal serum estradiol and the intellectual development of children conceived via ART.

## Materials and methods

### Study design and patient recruitment

**Fig. 1:**
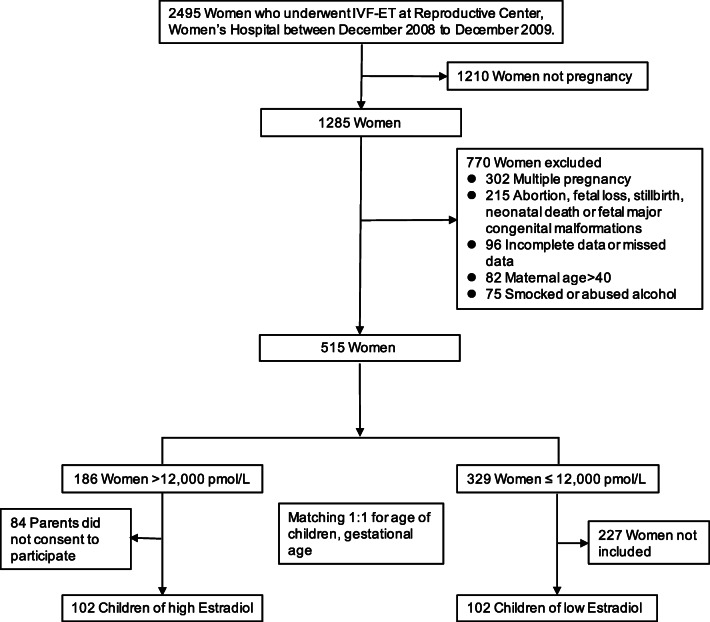
Flow chart of the clinical study

Women who underwent in vitro fertilization (IVF) at the reproductive medicine center, Women’s Hospital, Zhejiang University, participated in this study from December 2008 through December 2009. Patients were included if they underwent IVF and fresh embryo transfer, and the maternal estradiol concentration on the day of hCG (human chorionic gonadotropin) administration (hCG day) was > 12,000 pmol/L, which was the median value for estradiol associated with fresh embryo transfer in our previous study [16]. We used the following as exclusion criteria: women who declined to participate; incomplete medical data or missing data (e.g., maternal serum estradiol); previous diagnosis of a psychiatric or psychological disease; multiple pregnancy; mothers who were > 40 years old at the time of IVF; smoking or alcohol abuse (> 14 drinks/week for women and > 21 drinks/week for men) by either parent within 3 months before IVF; abortion; fetal loss; stillbirth; neonatal death; and major congenital malformations in the offspring. We created a 1:1 matched control cohort composed of IVF women whose estradiol concentration on hCG day was ≤ 12,000 pmol/L and their children. The gestational age (within 1 week) and the age of children (within 6 months) were chosen as matching factors that influence cognitive development according to previous studies [18–20]. A total of 102 children born from mothers having > 12,000 pmol/L serum estradiol on hCG day (high group) and 102 children born from mothers having ≤ 12,000 pmol/L serum estradiol on hCG day (low group) were included in the analysis (Fig. 1).

Parental information and ART characteristics were collected through parental interviews and review of medical records. The height and weight of the children were measured during their assessments. Maternal and paternal education levels were classified into three groups: low (up through completion of middle school), middle (completion of high school), and high (completion of college or above). The Ethics Committee of Women’s Hospital, School of Medicine, Zhejiang University approved the study (reference number: 20120003). Signed informed consent forms were obtained from the parents of these children. The investigation was performed according to the principles outlined in the Declaration of Helsinki and was retrospectively registered in the Chinese Clinical Trial Registry (ChiCTR-SOC-16009555).

### Evaluation of intellectual development

The intellectual development of the 4- to 7-year-old children was assessed according to the Chinese version of the Wechsler Preschool and Primary Scale of Intelligence (WPPSI) [21]. All children completed ten individual tests, including five verbal tests and five performance tests. The verbal tests included Information (I), Vocabulary (V), Arithmetic (A), Sorting (S) and Comprehension (C), whereas the performance tests included Animal Pegs (AP), Picture Completion (PC), Mazes (M), Object Assembly (OA), and Block Design (BD). The raw score from each intelligence subtest was converted to an age-scaled score of intelligence quotient (IQ) according to a nationally standardized norm of China [22], and the following three IQ types were calculated: Verbal IQ (VIQ, transformed from the sum of scaled verbal scores), Performance IQ (PIQ, transformed from the sum of scaled performance scores), and Full scale IQ (FSIQ, transformed from the total of all scaled scores). The mean score and standard deviation for a normal population is 100 ± 15 [22]. Moreover, three other IQ factors were measured: the Verbal Comprehension Factor (VC: I + V + C + S), Perceptual Organization Factor (PO: PC + BD + OA), and Memory/Caution Factor (M/C: A + AP + MA) [23]. Intelligence tests are routinely given to children conceived via ART, and the tests were administered by trained professionals who were blinded to the study grouping.

### Statistical analysis

All statistical analyses were performed using SPSS (version 22.0). Data with a probability score of *p* < 0.05 were considered statistically significant. All continuous variables were analyzed for distribution normality using the Kolmogorov-Smirnov test and Q-Q plot. Student’s *t* test or Mann-Whitney *U* test was performed for the continuous variable. Categorical variables were compared using two-tailed chi-square tests. The mean differences were adjusted with a linear regression model to control for certain confounders including birth weight, sex of the child, parents’ education level, and maternal variables including polycystic ovarian syndrome (PCOS), OHSS, gestational diabetes (GDM), preeclampsia (PE), and intrahepatic cholestasis of pregnancy (ICP). Meanwhile, partial correlation analysis adjusted for sex of the child, parents’ education level, and maternal variables was adopted to investigate the relationship between maternal serum estradiol level and intellectual development of their children. Sample size was calculated before the study on the basis of our previously reported IQs of IVF children [16]. Considering score differences of 5 in FSIQ between high and low estradiol subjects (SD = 10; power *N* = 0.9; α = 5%; sampling ratio = 1), 84 cases in each group were required.

## Results

We recruited 204 women and their 204 children who completed the IQ tests. Table 1 shows the clinical information for the children and parents. The age, gender, height, weight, and body mass index of children at follow-up time were similar between the low and high groups. The percentage of preterm births (gestational age < 37 weeks) among the overall study population was 21.1% (43/204), but no significant difference was noted between the two groups. The birth weight of children in the high group was lower than that in the low group, which is consistent with our published findings [11]. Moreover, OHSS incidence rates in the high group were slightly higher than those in the low group, which may be a result of the estradiol inclusion criteria. The parental data were similar between the two groups except for maternal serum estradiol level on hCG day.

**Table 1:** Clinical characteristics of children and parents in the low and high serum estradiol groups

Characteristic^a^	Low (*n* = 102)	High (*n* = 102)	*p* value^b^
Children			
Age at follow-up, years	4.95 (0.27)	5.02 (0.42)	0.830^c^
Sex, male (%)	59 (57.8)	56 (54.9)	0.672
Height at follow-up, cm	111.8 (4.36)	111.1 (4.50)	0.251
Weight at follow-up, kg	19.0 (2.63)	18.6 (2.38)	0.415^c^
BMI, kg/m^2^	15.2 (1.46)	15.1 (1.50)	0.601^c^
Delivery data			
Gestational age at birth, week	37.8 (2.37)	37.5 (1.86)	0.451
Preterm birth (%)	18 (17.6)	25 (24.5)	0.230
Birth weight, g	3153 (700)	2978 (556)	0.049
Cesarean section (%)	53 (52.0)	60 (58.8)	0.324
Apgar < 7 at 5 min	4 (3.9)	2 (2.0)	0.407
Respiratory distress syndrome (%)	1 (1.0)	0 (0.0)	0.316
Parents			
Maternal estradiol on day of hCG administration, pmol/L	9769 (1588)	15,081 (2901)	< 0.0001^c^
Maternal age, years	30.0 (3.23)	29.4 (3.44)	0.207
Nulliparous (%)	84 (82.4)	80 (78.4)	0.481
PCOS (%)	21 (20.6)	27 (26.5)	0.322
OHSS (%)	4 (3.9)	11 (10.8)	0.060
Gestational diabetes (%)	19 (18.6)	20 (19.6)	0.859
Preeclampsia (%)	5 (4.9)	7 (6.9)	0.552
Intrahepatic cholestasis of pregnancy (%)	5 (4.9)	10 (9.8)	0.180
Maternal education level (%)^d^			
Low	17 (16.7)	10 (9.8)	0.072
Middle	33 (32.4)	48 (47.1)	
High	52 (51.0)	44 (43.1)	
Type of infertility, primary (%)	70 (68.6)	65 (63.7)	0.459
Paternal age, years	32.5 (4.90)	32.5 (5.58)	0.995
Paternal education level (%)^d^			
Low	11 (10.8)	16 (15.7)	0.227
Middle	30 (29.4)	37 (36.3)	
High	61 (59.8)	49 (48.0)	

*OHSS*, ovarian hyper-stimulation syndrome; *BMI*, body mass index; *PCOS*, polycystic ovarian syndrome

^a^Data are expressed as the mean (SD) or the number of participants (percent)

^b^The *p* values were calculated by the Student’s *t* test or chi-square test unless otherwise specified

^c^The *p* values were calculated by the Mann-Whitney *U* test

^d^Maternal and paternal education levels were classified as low (up through completion of middle school), middle (completion of high school), and high (completion of college or above)

Intellectual development was evaluated with WPPSI (Table 2). Children from the high group scored lower in VIQ (− 4.9, 95% confidence interval, CI, − 8.5 to − 1.3) and verbal comprehension (− 3.3, 95% CI − 5.6 to − 1.0), but no statistical difference was observed in PIQ, perceptual organization, and memory/caution. Owing to the lower scores in VIQ, children of the high group were 3.9 (95% CI − 7.1 to − 0.8) lower than that of the low group in FSIQ. To solidify the results, we further analyzed the data with a linear regression model to control for potential confounding factors and to assess the effects of prognostic factors. We adjusted for birth weight, sex of the child, parents’ education level, and maternal variables (PCOS, OHSS, GDM, PE, and ICP). After adjustment, VIQ (− 4.5, 95% CI − 8.3 to − 0.7) and verbal comprehension (− 2.9, 95% CI − 5.3 to − 0.5) of the high group children were still significantly lower than the corresponding values for the low group children. Details of subtests are shown in Table 3. The main differences between the two groups occurred in information, vocabulary, and sorting subtest.

**Table 2:** IQ of children exposed to a low or high level of estradiol in utero

Characteristic^a^	Low (*n* = 102)	High (*n* = 102)	Mean difference (95% CI) unadjusted	Mean difference (95% CI) adjusted^b^
VIQ	101.7 (11.7)	96.8 (14.3)	− 4.9 (− 8.5 to − 1.3) **	− 4.5 (− 8.3 to − 0.7) *
PIQ	114.5 (10.4)	112.3 (11.4)	− 2.1 (− 5.2 to 0.9)	− 2.2 (− 5.3 to 1.0)
FIQ	108.7 (9.9)	104.8 (12.7)	− 3.9 (− 7.1 to − 0.8) *	− 3.7 (− 7.0 to −0.4) *
Verbal Comprehension	42.1 (7.4)	38.8 (9.0)	− 3.3 (− 5.6 to − 1.0) **	− 2.9 (− 5.3 to − 0.5) *
Perceptual Organization	33.3 (5.0)	32.2 (5.4)	− 1.1 (− 2.5 to 0.3)	− 1.1 (− 2.5 to 0.4)
Memory/caution	36.2 (5.0)	35.5 (5.2)	− 0.6 (− 2.1 to 0.8)	− 0.8 (− 2.2 to 0.7)

*VIQ*, Verbal IQ; *PIQ*, Performance IQ; *FIQ*, Full IQ

*p < 0.05; **p < 0.01

^a^Data are expressed as the mean (SD) or mean difference (95% confidence interval)

^b^Adjusted for birth weight, gender of child, maternal education level, paternal education level, and maternal variables (including OHSS, PCOS, ICP, GDM, and PE)

**Table 3:** Subtest scores of the low and high groups

Characteristic^a^	Low (*n* = 102)	High (*n* = 102)	Mean difference (95% CI)	Mean difference (95% CI) adjusted^b^	Effect sizes (Cohen’s d)
Verbal test					
Information	9.6 (2.8)	8.5 (3.1)	− 1.1 (− 1.9 to − 0.3) *	− 1.0 (− 1.9 to − 0.2) *	0.339
Vocabulary	11.1 (2.3)	10.3 (2.6)	− 0.8 (− 1.5 to − 0.1) *	− 0.7 (− 1.4 to − 0.1) *	0.326
Arithmetic	9.2 (2.7)	8.9 (2.7)	− 0.2 (− 0.9 to 0.5)	− 0.3 (− 1.1 to 0.5)	0.111
Sorting	11.3 (2.6)	10.4 (2.5)	− 0.9 (− 1.5 to − 0.2) *	− 0.8 (− 1.5 to − 0.1) *	0.353
Comprehension	10.1 (2.5)	9.6 (3.4)	− 0.5 (− 1.3 to 0.2)	− 0.3 (− 1.2 to 0.5)	0.168
Performance tests					
Animal Pegs	13.2 (1.7)	13.0 (2.0)	− 0.3 (− 0.8 to 0.2)	− 0.3 (− 0.8 to 0.2)	0.108
Picture Completion	10.1 (2.6)	9.7 (2.5)	− 0.4 (− 1.1 to 0.3)	− 0.4 (− 1.1 to 0.4)	0.157
Mazes	13.8 (2.8)	13.6 (2.6)	− 0.2 (− 0.9 to 0.5)	− 0.2 (− 1.0 to 0.6)	0.074
Object Assembly	11.4 (2.3)	10.8 (2.2)	− 0.5 (− 1.2 to 0.1)	− 0.5 (− 1.1 to 0.2)	0.267
Block Design	11.9 (2.1)	11.7 (2.4)	− 0.2 (− 0.8 to 0.4)	− 0.2 (− 0.9 to 0.4)	0.089

*p < 0.05

^a^Data are expressed as the mean (SD) or mean difference (95% confidence interval)

^b^Adjusted for birth weight, gender of child, maternal education level, paternal education level, and maternal variables (including OHSS, PCOS, ICP, GDM, and PE)

**Fig. 2:**
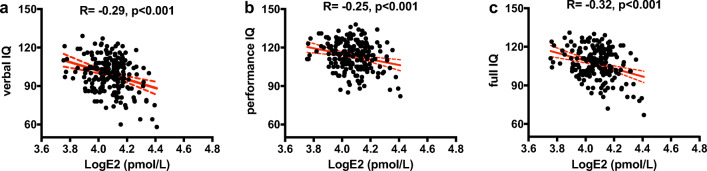
Correlation between maternal serum estradiol levels on hCG day and offspring IQ scores (*n* = 204). A negative linear correlation between maternal serum estradiol levels on hCG day and (**a**) Verbal IQ, (**b**) Performance IQ, and (**c**) Full IQ is represented by the solid red line. The red dotted lines indicate the 95% confidence intervals. The partial correlation analysis was adjusted for sex of the children, parental education level, and maternal variables (PCOS, OHSS, GDM, PE, and ICP). E2, estradiol

Upon determining that maternal serum estradiol level on hCG day correlated positively with maternal serum estradiol levels at 4 and 8 weeks of gestation [11], we performed partial correlation analysis between the serum estradiol level of the mother on hCG day and the intellectual development of her offspring to investigate the association between maternal serum estradiol and offspring IQ. In this analysis, we adjusted for sex of the child, parents’ education level, and maternal variables (PCOS, OHSS, GDM, PE, and ICP). The results revealed a negative correlation between the logarithm of the maternal serum estradiol level and the VIQ (*R* = − 0.29, *p* < 0.001), PIQ (*R* = − 0.25, *p* < 0.001), and FSIQ (*R* = − 0.32, *p* < 0.001) of the children (Fig. 2).

## Discussion and conclusion

The issue of whether ART can affect the cognitive development of the resulting children has long attracted attention. Most studies have found comparable cognitive development between ART-conceived and spontaneously conceived children [24, 25]. Our previous study showed that children born to OHSS mothers had lower IQs than non-OHSS IVF children, and this difference was associated with high maternal serum estradiol. However, it remains unknown whether IQ is affected in children born to mothers with high serum estradiol. In this study, using the mean value (12,000 pmol/L) of estradiol associated with fresh embryo transfer as a reference [16], we recruited children whose mothers had high serum estradiol (> 12,000 pmol/L) or low serum estradiol (≤ 12,000 pmol/L) on hCG day. Children from the high group had had lower verbal ability scores (VIQ and verbal comprehension) than children from the low group. Owing to the lower scores for VIQ, FSIQ scores were statistically different between the two groups. However, the mean FSIQ scores of the two groups were still above 100 and within the normal range. Moreover, partial correlation analysis revealed that the IQ of the children negatively correlated with maternal serum estradiol level. These results suggest that exposure to a high estradiol environment during early embryonic development may negatively associate with verbal ability development.

Abnormalities in the prenatal environment can increase the risk of chronic diseases in later life, including diabetes, cardiovascular diseases, and endocrine dysfunction [12, 26, 27]. The theory that the root of adult diseases can lie in early fetal life events was termed the “Barker Hypothesis” in 1995 [28], and increasing evidence supporting this hypothesis has been reported in the intervening years [29–31]. Moreover, many reports have described how pregnancy events may influence the brain development of offspring. Obstetric complications, such as GDM [32, 33] and PE [34], can correlate with impaired cognitive abilities and psychiatric disorders in offspring. Prenatal exposure to excess hormones can cause abnormal developmental of the brain in offspring. We previously reported that the ART complication of OHSS has adverse effects on the IQ of children who also experienced prenatal exposure to high estradiol [16]. In addition, animal models have shown that exposure to excess glucocorticoids during fetal development results in increased anxiety and depression-like behavior [35, 36]; in humans, exposure can alter the function of the hypothalamic-pituitary-adrenal axis [37]. Prenatal exposure to higher levels of hormones of the steroidogenic pathway, including the androgens testosterone and androstenedione, can modify brain development of children and mediate a higher risk of autism spectrum disorder [38]. Moreover, children of women with PCOS appear to have a higher risk of developing autism spectrum disorder, which is associated with exposure to excessive maternal androgens [39]. In the current study, we accounted for these maternal variables to adjust for their effect on the IQ scores of children, as well as other confounders including birth weight [40, 41], paternal education level, and gender of children.

Few studies have associated brain development and behavioral consequences with exposure to high estradiol. Disruption of estrogen signaling in the developing cerebellum of mice reduces Purkinje cell growth in both males and females and reduces social behavior in male mice [42]. Recently, researchers found that elevated levels of estrogen (estradiol, estriol, estrone) in amniotic fluid contribute to autism in children, with estradiol levels being the most significant predictor of autism likelihood in univariate logistic regression models [43]. Autism spectrum disorder refers to a neurodevelopmental condition associated with abnormal verbal development, social interactions, and behavioral complications. In our study, verbal ability in the high prenatal estradiol group was significantly lower than that in the low group. To our knowledge, this is the first reported association of an estrogen with decreased verbal ability in ART children. This result supports the need for further studies on behavioral disorders in ART children who experienced high maternal estradiol, with the use of appropriate scales such as the Childhood Autism Rating Scale [44].

There are several limitations and considerations with regard to the present study. First, the correlation between maternal serum estradiol and amniotic fluid estradiol is not entirely clear [45]. Second, apart from the education level of the parents, other socioeconomic data (e.g., family income, city, country) that may influence the IQ of children were not available in our study. Also, the evaluated children were 4 to 7 years old, and their intelligence levels were still in a stage of rapid development. Finally, a long-term follow-up with more samples and an appropriate rating scale is necessary to validate our findings. In conclusion, our data demonstrate that prenatal exposure to excessive maternal serum estradiol in utero may diminish the verbal ability of ART children. These findings reveal a new field of study in ART long-term safety and provide an incentive to improve ART strategy to prevent excessive estradiol exposure.

## References

[1] Newbold RR, Padilla-Banks E, Snyder RJ, Jefferson WN (2007). Perinatal exposure to environmental estrogens and the development of obesity. Mol Nutr Food Res.

[2] Ng SF, Lin RC, Laybutt DR, Barres R, Owens JA, Morris MJ (2010). Chronic high-fat diet in fathers programs beta-cell dysfunction in female rat offspring. Nature..

[3] Radford EJ, Ito M, Shi H, Corish JA, Yamazawa K, Isganaitis E, Seisenberger S, Hore TA, Reik W, Erkek S, Peters AHFM, Patti ME, Ferguson-Smith AC (2014). In utero undernourishment perturbs the adult sperm methylome and intergenerational metabolism. Science..

[4] Wei Y, Yang CR, Wei YP, Zhao ZA, Hou Y, Schatten H, Sun QY (2014). Paternally induced transgenerational inheritance of susceptibility to diabetes in mammals. Proc Natl Acad Sci U S A.

[5] Jimenez-Chillaron JC, Isganaitis E, Charalambous M, Gesta S, Pentinat-Pelegrin T, Faucette RR, Otis JP, Chow A, Diaz R, Ferguson-Smith A, Patti ME (2009). Intergenerational transmission of glucose intolerance and obesity by in utero undernutrition in mice. Diabetes..

[6] Sasaki H, Matsui Y (2008). Epigenetic events in mammalian germ-cell development: reprogramming and beyond. Nat Rev Genet.

[7] Smallwood SA, Tomizawa S, Krueger F, Ruf N, Carli N, Segonds-Pichon A, Sato S, Hata K, Andrews SR, Kelsey G (2011). Dynamic CpG island methylation landscape in oocytes and preimplantation embryos. Nat Genet.

[8] Smith ZD, Chan MM, Mikkelsen TS, Gu H, Gnirke A, Regev A, Meissner A (2012). A unique regulatory phase of DNA methylation in the early mammalian embryo. Nature..

[9] Weaver IC, Cervoni N, Champagne FA, D'Alessio AC, Sharma S, Seckl JR (2004). Epigenetic programming by maternal behavior. Nat Neurosci.

[10] Jarvela IY, Pelkonen S, Uimari O, Makikallio K, Puukka K, Ruokonen A, Tekay A, Martikainen H (2014). Controlled ovarian hyperstimulation leads to high progesterone and estradiol levels during early pregnancy. Hum Reprod.

[11] Hu XL, Feng C, Lin XH, Zhong ZX, Zhu YM, Lv PP, Lv M, Meng Y, Zhang D, Lu XE, Jin F, Sheng JZ, Xu J, Huang HF (2014). High maternal serum estradiol environment in the first trimester is associated with the increased risk of small-for-gestational-age birth. J Clin Endocrinol Metab.

[12] Xu GF, Zhang JY, Pan HT, Tian S, Liu ME, Yu TT, Li JY, Ying WW, Yao WM, Lin XH, Lv Y, Su WW, Ye XQ, Zhang FH, Pan JX, Liu Y, Zhou CL, Zhang D, Liu XM, Zhu YM, Sheng JZ, Huang HF (2014). Cardiovascular dysfunction in offspring of ovarian-hyperstimulated women and effects of estradiol and progesterone: a retrospective cohort study and proteomics analysis. J Clin Endocrinol Metab.

[13] Lv PP, Meng Y, Lv M, Feng C, Liu Y, Li JY, Yu DQ, Shen Y, Hu XL, Gao Q, Dong S, Lin XH, Xu GF, Tian S, Zhang D, Zhang FH, Pan JX, Ye XQ, Liu ME, Liu XM, Sheng JZ, Ding GL, Huang HF (2014). Altered thyroid hormone profile in offspring after exposure to high estradiol environment during the first trimester of pregnancy: a cross-sectional study. BMC Med.

[14] Lv PP, Tian S, Feng C, Li JY, Yu DQ, Jin L, Shen Y, Yu TT, Meng Y, Ding GL, Jin M, Chen XJ, Sheng JZ, Zhang D, Huang HF (2016). Maternal high estradiol exposure is associated with elevated Thyroxine and Pax8 in mouse offspring. Sci Rep.

[15] Meng Y, Lv PP, Ding GL, Yu TT, Liu Y, Shen Y, Hu XL, Lin XH, Tian S, Lv M, Song Y, Guo MX, Ke ZH, Xu H, Sheng JZ, Shi FT, Huang HF (2015). High maternal serum estradiol levels induce dyslipidemia in human newborns via a hepatic HMGCR estrogen response element. Sci Rep.

[16] Xu GF, Zhou CL, Xiong YM, Li JY, Yu TT, Tian S, Lin XH, Liao Y, Lv Y, Zhang FH, Liu ZW, Shi YY, Shen Y, Sha J, Zhang D, Zhu YM, Sheng JZ, Huang HF (2017). Reduced intellectual ability in offspring of ovarian hyperstimulation syndrome: a cohort study. EBioMedicine..

[17] Gore AC, Martien KM, Gagnidze K, Pfaff D (2014). Implications of prenatal steroid perturbations for neurodevelopment, behavior, and autism. Endocr Rev.

[18] McCarton CM, Wallace IF, Divon M, Vaughan HG (1996). Cognitive and neurologic development of the premature, small for gestational age infant through age 6: comparison by birth weight and gestational age. Pediatrics..

[19] Bhutta AT, Cleves MA, Casey PH, Cradock MM, Anand KJ (2002). Cognitive and behavioral outcomes of school-aged children who were born preterm: a meta-analysis. JAMA..

[20] Twilhaar ES, Wade RM, de Kieviet JF, van Goudoever JB, van Elburg RM, Oosterlaan J (2018). Cognitive outcomes of children born extremely or very preterm since the 1990s and associated risk factors: a meta-analysis and meta-regression. JAMA Pediatr.

[21] Liu J, Yang H, Li L, Chen T, Lynn R (2012). An increase of intelligence measured by the WPPSI in China, 1984-2006. Intelligence..

[22] Guo BL, Aveyard P, Dai XY (2009). The Chinese intelligence scale for young children testing factor structure and measurement invariance using the framework of the Wechsler intelligence tests. Educ Psychol Meas.

[23] Yu B, Kong F, Peng M, Ma H, Liu N, Guo Q (2012). Assessment of memory/attention impairment in children with primary nocturnal enuresis: a voxel-based morphometry study. Eur J Radiol.

[24] Bay B, Mortensen EL, Kesmodel US (2013). Assisted reproduction and child neurodevelopmental outcomes: a systematic review. Fertil Steril.

[25] Barbuscia A, Mills MC (2017). Cognitive development in children up to age 11 years born after ART-a longitudinal cohort study. Hum Reprod.

[26] Vuguin PM, Hartil K, Kruse M, Kaur H, Lin CL, Fiallo A (2013). Shared effects of genetic and intrauterine and perinatal environment on the development of metabolic syndrome. PLoS One.

[27] Martinez-Arguelles DB, Campioli E, Culty M, Zirkin BR, Papadopoulos V (2013). Fetal origin of endocrine dysfunction in the adult: the phthalate model. J Steroid Biochem Mol Biol.

[28] Paneth N, Susser M (1995). Early origin of coronary heart disease (the “Barker hypothesis”). Bmj..

[29] Fannon SA, Vidaver RM, Marts SA (2002). Early encounters, lifetime effects: hormones in the intrauterine environment. Trends Endocrinol Metab.

[30] Heijmans BT, Tobi EW, Stein AD, Putter H, Blauw GJ, Susser ES, Slagboom PE, Lumey LH (2008). Persistent epigenetic differences associated with prenatal exposure to famine in humans. Proc Natl Acad Sci U S A.

[31] Huang H-F, Sheng J-Z. Gamete and embryo-fetal origins of adult diseases: Springer; 2014.

[32] Fraser A, Nelson SM, Macdonald-Wallis C, Lawlor DA (2012). Associations of existing diabetes, gestational diabetes, and glycosuria with offspring IQ and educational attainment: the Avon Longitudinal Study of Parents and Children. Exp Diabetes Res.

[33] Xiang AH (2017). Association of maternal diabetes with autism in offspring. JAMA..

[34] Walker CK, Krakowiak P, Baker A, Hansen RL, Ozonoff S, Hertz-Picciotto I (2015). Preeclampsia, placental insufficiency, and autism spectrum disorder or developmental delay. JAMA Pediatr.

[35] Hossain A, Hajman K, Charitidi K, Erhardt S, Zimmermann U, Knipper M, Canlon B (2008). Prenatal dexamethasone impairs behavior and the activation of the BDNF exon IV promoter in the paraventricular nucleus in adult offspring. Endocrinology..

[36] Bale TL (2005). Sensitivity to stress: dysregulation of CRF pathways and disease development. Horm Behav.

[37] Wyrwoll CS, Holmes MC (2012). Prenatal excess glucocorticoid exposure and adult affective disorders: a role for serotonergic and catecholamine pathways. Neuroendocrinology..

[38] Baron-Cohen S, Auyeung B, Norgaard-Pedersen B, Hougaard DM, Abdallah MW, Melgaard L (2015). Elevated fetal steroidogenic activity in autism. Mol Psychiatry.

[39] Kosidou K, Dalman C, Widman L, Arver S, Lee BK, Magnusson C, Gardner RM (2016). Maternal polycystic ovary syndrome and the risk of autism spectrum disorders in the offspring: a population-based nationwide study in Sweden. Mol Psychiatry.

[40] Gu H, Wang L, Liu L, Luo X, Wang J, Hou F, Nkomola PD, Li J, Liu G, Meng H, Zhang J, Song R (2017). A gradient relationship between low birth weight and IQ: a meta-analysis. Sci Rep.

[41] Upadhyay RP, Naik G, Choudhary TS, Chowdhury R, Taneja S, Bhandari N, Martines JC, Bahl R, Bhan MK (2019). Cognitive and motor outcomes in children born low birth weight: a systematic review and meta-analysis of studies from South Asia. BMC Pediatr.

[42] Hoffman JF, Wright CL, McCarthy MM (2016). A critical period in Purkinje cell development is mediated by local estradiol synthesis, disrupted by inflammation, and has enduring consequences only for males. J Neurosci.

[43] Baron-Cohen S, Tsompanidis A, Auyeung B, Norgaard-Pedersen B, Hougaard DM, Abdallah M, et al. Foetal oestrogens and autism. Mol Psychiatry. 2019. 10.1038/s41380-019-0454-9. 10.1038/s41380-019-0454-9 PMC7577840 31358906

[44] Thabtah F, Peebles D. Early autism screening: a comprehensive review. Int J Environ Res Public Health. 2019;16(18). 10.3390/ijerph16183502. 10.3390/ijerph16183502 PMC6765988 31546906

[45] van de Beek C, Thijssen JH, Cohen-Kettenis PT, van Goozen SH, Buitelaar JK (2004). Relationships between sex hormones assessed in amniotic fluid, and maternal and umbilical cord serum: what is the best source of information to investigate the effects of fetal hormonal exposure?. Horm Behav.

